# Study on the Coordination Structure of Pt Sorbed on Bacterial Cells Using X-Ray Absorption Fine Structure Spectroscopy

**DOI:** 10.1371/journal.pone.0127417

**Published:** 2015-05-21

**Authors:** Kazuya Tanaka, Naoko Watanabe

**Affiliations:** Institute for Sustainable Sciences and Development, Hiroshima University, Higashi-Hiroshima, Japan; Banaras Hindu University, INDIA

## Abstract

Biosorption has been intensively investigated as a promising technology for the recovery of precious metals from solution. However, the detailed mechanism responsible for the biosorption of Pt on a biomass is not fully understood because of a lack of spectroscopic studies. We applied X-ray absorption fine structure spectroscopy to elucidate the coordination structure of Pt sorbed on bacterial cells. We examined the sorption of Pt(II) and Pt(IV) species on bacterial cells of *Bacillus subtilis* and *Shewanella putrefaciens* in NaCl solutions. X-ray absorption near-edge structure and extended X-ray absorption fine structure (EXAFS) of Pt-sorbed bacteria suggested that Pt(IV) was reduced to Pt(II) on the cell’s surface, even in the absence of an organic material as an exogenous electron donor. EXAFS spectra demonstrated that Pt sorbed on bacterial cells has a fourfold coordination of chlorine ions, similar to PtCl_4_^2-^, which indicated that sorption on the protonated amine groups of the bacterial cells. This work clearly demonstrated the coordination structure of Pt sorbed on bacterial cells. The findings of this study will contribute to the understanding of Pt biosorption on biomass, and facilitate the development of recovery methods for rare metals using biosorbent materials.

## Introduction

The platinum group metals (PGM), (Pd, Pt, and Rh), are of great interest as catalysts in various industries, in particular, their obligatory utilization in automotive catalytic converters, which is necessary to effectively reduce the hydrocarbons, CO, and NO_x_ emitted in exhaust gas [1]. However, the natural abundance of PGM is low, and even ore minerals only contain approximately 3 ppm of Pt [2], indicating a limited availability of PGM from natural metal resources. Consequently, the recovery of PGM from industrial waste is of great economic importance in order to satisfy the increasing demand for these metals. Currently, the main large-scale industrial recovery processes used are based on hydro- or pyrometallurgical techniques, but these conventional technologies generate waste solution that still contains residual precious metals. In this context, the recovery of PGM from aqueous and waste solutions is an economically attractive subject.

It is necessary to develop a low-cost and eco-friendly technique applicable to the recovery of precious metals from wastewater that generates little secondary waste [3]. Biomass has the merit of being relatively inexpensive and is scalable to large volumes; therefore, the application of microbial activity and biomass techniques, such as biosorption and bioreduction, can satisfy such requirement. Bioreduction, such as Pd(II) to Pd(0) and Pt(IV) to Pt(0), is well known as a useful technique for the recovery of PGM from aqueous solutions using sulfate-reducing bacteria [4,5]. The utilization and efficiency of microorganisms such as bacteria and fungi in the biosorption of PGM have been also investigated as a promising technology for the recovery of PGM from solution [6–11].

Biosorption is considered to be a metabolism-independent process that occurs at the interface between a cell and a solution because the fact that the properties of types of inactive or dead microbial biomass materials allows them to bind and concentrate metal ions from aqueous solutions [6,9]. Various functional groups, such as carboxyl, hydroxyl, amine, and phosphate groups, are known to be present on the surface (cell wall) of bacterial cells, and have been found to be responsible for metal biosorption [9,12]. Positively charged ions of cationic metal species are bound to deprotonated carboxyl and phosphate groups [13,14]. In contrast, amine groups, which are protonated in low pH solutions, are preferred for binding anionic species through electrostatic interactions [15–17].

Platinum can exist in solution as dissolved species in two oxidation states: Pt(II) and Pt(IV), both of which form chloro-complexes, such as PtCl_4_^2-^ and PtCl_6_^2-^, particularly in the presence of Cl^-^ ions. Using X-ray photoelectron spectroscopy (XPS), Won et al. [11] suggested that biosorption of Pt (PtCl_6_^2-^) occurred from the electrostatic attraction of anionic Pt chloride complexes to protonated amine groups on the biomass surface. However, XPS does not directly show the coordination structure of Pt bonded to the functional groups on a cell’s surface. Therefore, the details of the coordination structure of Pt sorbed on microbial cells and a biomass surface are not fully understood due to a scarcity of studies using other spectroscopic methods.

X-ray absorption fine structure (XAFS) spectroscopy is useful for observing the local coordination structure of an element in various forms. XAFS is divided into the two different energy regions: X-ray absorption near-edge structure (XANES) and extended X-ray absorption fine structure (EXAFS). XANES is useful for determining the oxidation state of a target element. EXAFS reveals information on the local coordination structure around a target atom, such as its coordination number (CN) and the interatomic distance to surrounding elements. In previous work, we successfully applied XAFS to studies on the biosorption of trace elements on microbial cells [14,18,19].

Many researchers mainly focused on the sorption efficiency and capacity of the biomass for metal ions in a technological sense, and the details of the biosorption mechanism have not extensively investigated. In this study, we used XAFS spectroscopy to clarify the coordination structure of Pt sorbed on bacterial cells. The findings of this study will contribute to the understanding of Pt biosorption on biomass, and facilitate the development of recovery methods for rare metals including PGM using biosorbent materials.

## Materials and Methods

### Preparation of bacterial cells

We used *Bacillus subtilis* (IAM 1069) and *Shewanella putrefaciens* (NBRC3908) in our Pt sorption experiments as model Gram-positive and Gram-negative bacteria, respectively. First, both *B*. *subtilis* and *S*. *putrefaciens* were precultured in 50 mL of a sterilized liquid medium containing 30 g L^-1^ of Tryptic soy broth (Bacto) at 30°C overnight. Then, precultured bacterial cells were inoculated into a new liquid medium, and again incubated at 30°C overnight. The bacterial cells were harvested using centrifugation, followed by washing three times with 0.01 mol L^-1^, 0.1 mol L^-1^, or 0.5 mol L^-1^ NaCl solution, corresponding to the conditions of the subsequent sorption experiments. The harvested cells were suspended in 0.01 mol L^-1^, 0.1 mol L^-1^, or 0.5 mol L^-1^ NaCl solution, and stored in a refrigerator at 4°C until used in the sorption experiments.

### Sorption experiments of Pt on bacterial cells

Sorption experiments of Pt(II) and Pt(IV) on bacterial cells were conducted in 0.01 mol L^-1^, 0.1 mol L^-1^, and 0.5 mol L^-1^ NaCl solutions. A stock solution containing 100 mg L^-1^ of Pt(II) or Pt(IV) was prepared from K_2_PtCl_4_ and K_2_PtCl_6_, respectively, by dissolution in a 1 mol L^-1^ HCl solution. The cell suspensions, prepared as described above, were placed in a polyethylene bottle, and then the desired concentration of NaCl solution was added to adjust the cell density to a value around 0.1 g L^-1^. The initial Pt concentration in the solutions was adjusted to 1 mg L^-1^ by addition of the Pt stock solution. After adjustment of the initial pH values to between 2 < pH < 4, the solutions were incubated at 25°C in a reciprocal shaker (approximately 100 strokes min^-1^) for a period of two weeks. In the experiments in a solution with an initial pH 4, the pH of the solution increased gradually with time, but no further adjustment to the pH was made.

Samples were taken from the solutions after given sorption time, and these were then filtered through a membrane filter having a pore size of 0.2 μm. The concentration of Pt in the filtrates was determined using an inductively-coupled plasma mass spectrometer (ICP-MS; Agilent7500, Agilent, USA). An internal Bi standard was added to each sample solution at a concentration of 1 ng mL^-1^. The signal intensities arising from ^194^Pt, ^195^Pt, and ^196^Pt were detected, and the Pt concentration was calculated from the average of the three isotopes. Platinum-sorbed bacterial cells, after a sorption time of 72 h, were collected using a 0.2 μm pore-size membrane filter for the XAFS measurements, as described in the next section. Here, we defined the sorption rate using the following the equation:

$$Sorption\;rate=\;\frac{{\text{[Pt]}}_{\text{init}}\;-\;{\text{[Pt]}}_{\text{fil}}}{{\text{[Pt]}}_{\text{init}}}\;\times \;100\;(\%)$$ (1)

where [Pt]_fil_ is the concentration of Pt in the filtrate, while [Pt]_init_ is the initial Pt concentration (i.e., 1 mg L^-1^). Cobelo-Garcia et al. [20] have reported on the sorption of Pd(II), Pt(IV), and Rh(III) onto various types of containers. We conducted control experiments without any cells, but no sorption on the walls of the container (i.e., the polyethylene bottle) was observed.

### XAFS measurements

Platinum L_III_-edge XAFS spectra were collected at Beamline 12C at the Photon Factory at KEK (Tsukuba, Japan). The storage ring of the Photon Factory operated at 2.5 GeV with a typical beam current of 450 mA. The broadband synchrotron radiation from the storage ring was monochromatized using a pair of Si (111) crystals to obtain the incident X-ray beam. The beam size at the sample position was about 1 mm × 1 mm. Energy was calibrated by assigning the peak maximum of Pt metal foil at 11.562 keV. Both K_2_PtCl_4_ and K_2_PtCl_6_ were measured as reference materials for the divalent and tetravalent species, respectively. The reagent powder of the Pt compounds was diluted with BN powder to a concentration of 2 wt% Pt, followed by homogenization, and this was pressed to form a disk with a thickness appropriate for XAFS measurements in the transmission mode. Solutions with a concentration of 1000 mg L^-1^ Pt(II) and Pt(IV) in a 1 mol L^-1^ HCl solution were also prepared as reference solutions, where the PtCl_4_^2-^ and PtCl_6_^2-^ chloro-complexes were the dominant Pt species. XAFS spectra of the reference solutions and the Pt-sorbed cell samples were collected in the fluorescence mode. The fluorescence yield of each sample was monitored using a 19-element Ge solid-state detector (SSD). The dead time for SSD counting was corrected according to the methods of Nomura [21]. All the measurements were carried out at room temperature. The reference materials and the nondried Pt-sorbed bacterial cells were placed in a polyethylene bag for the XAFS measurements. Multiple scans for bacterial cells gave identical spectra to one another, indicating no significant radiation damage occurred during data acquisition.

Data analysis of the EXAFS spectra was carried out using the REX2000 v.2.5.9 software package (Rigaku Co. Ltd) [14]. After extraction of the EXAFS oscillations, the energy unit was converted from keV to Å^-1^ to produce the EXAFS function χ(k), where the photoelectron wave vector (k) is given by $\sqrt{2m\left(E-E_{0}/\hbar^{2}\right)}$, E is the energy of the incident X-ray, and E_0_ is the threshold energy for liberation of the photoelectron wave. The value of E_0_ was set at the edge inflection point for all the samples. The k^3^-weighted χ(k) function was Fourier transformed (FT) from k (Å^-1^) space into R (Å) space to give the radial structural function (RSF). The FT-EXAFS spectra of the RSF were back-transformed into k space for neighboring shells of interest in the RSF for spectral simulation. In these simulations, the theoretical EXAFS function was fitted to the back-transformed k^3^-weighted χ(k) functions using the backscattering amplitudes and phase shifts generated using the FEFF7.02 code [22,23]. The desired backscattering amplitudes and phase-shifts for the Pt—Cl bonds were extracted from the structures of K_2_PtCl_4_ and (NH_4_)_2_PtCl_4_.

## Results and Discussion

### Sorption of Pt on bacterial cells

**Fig 1:**
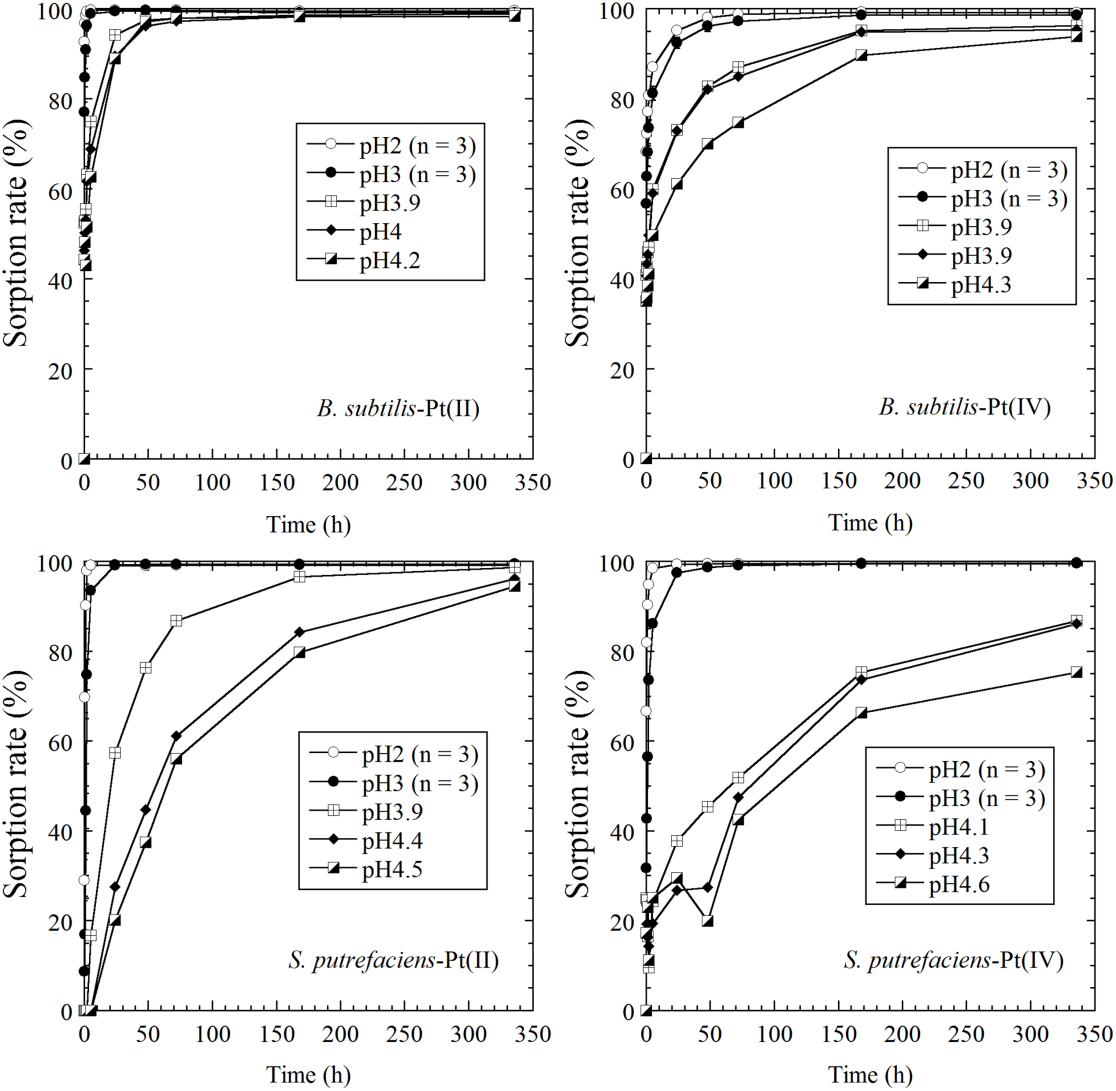
Time-course sorption of Pt(II) and Pt(IV) on *B*. *subtilis* and *S*. *putrefaciens* in 0.01 mol L^-1^ NaCl solution under different pH conditions. The pH values in the legend of each figure indicate the final pH values of the solution (i.e., after a period of two weeks). The average values of triplicate experiments at pH 2 and 3 are plotted, whereas the sorption rate for each experimental run is plotted at around pH 4 because of different final pH values.

Results of the time course sorption experiments in 0.01 mol L^-1^ NaCl solutions with different pH values are shown in Fig 1. Both *B*. *subtilis* and *S*. *putrefaciens* showed similar trends in the biosorption of Pt(II) and Pt(IV). Both of Pt(II) and Pt(IV) were sorbed onto the cells within two hours in solution at pH 2 and 3. The biosorption of Pt was slowed on increasing the pH, and the degree of Pt sorption was lower for higher pH values. The PtCl_4_^2-^ and PtCl_6_^2-^ chloro-complexes form the dominant Pt(II) and Pt(IV) species, respectively, in NaCl solutions [24,25]. It is well known that amine groups become protonated on decreasing the pH of a solution, offering positively charged binding sites (biomass-NH_3_^+^) for anionic species [15,17]. Therefore, the observed sorption behavior of Pt with changes in solution pH is reasonable and consistent with the observation of previous work.

**Fig 2:**
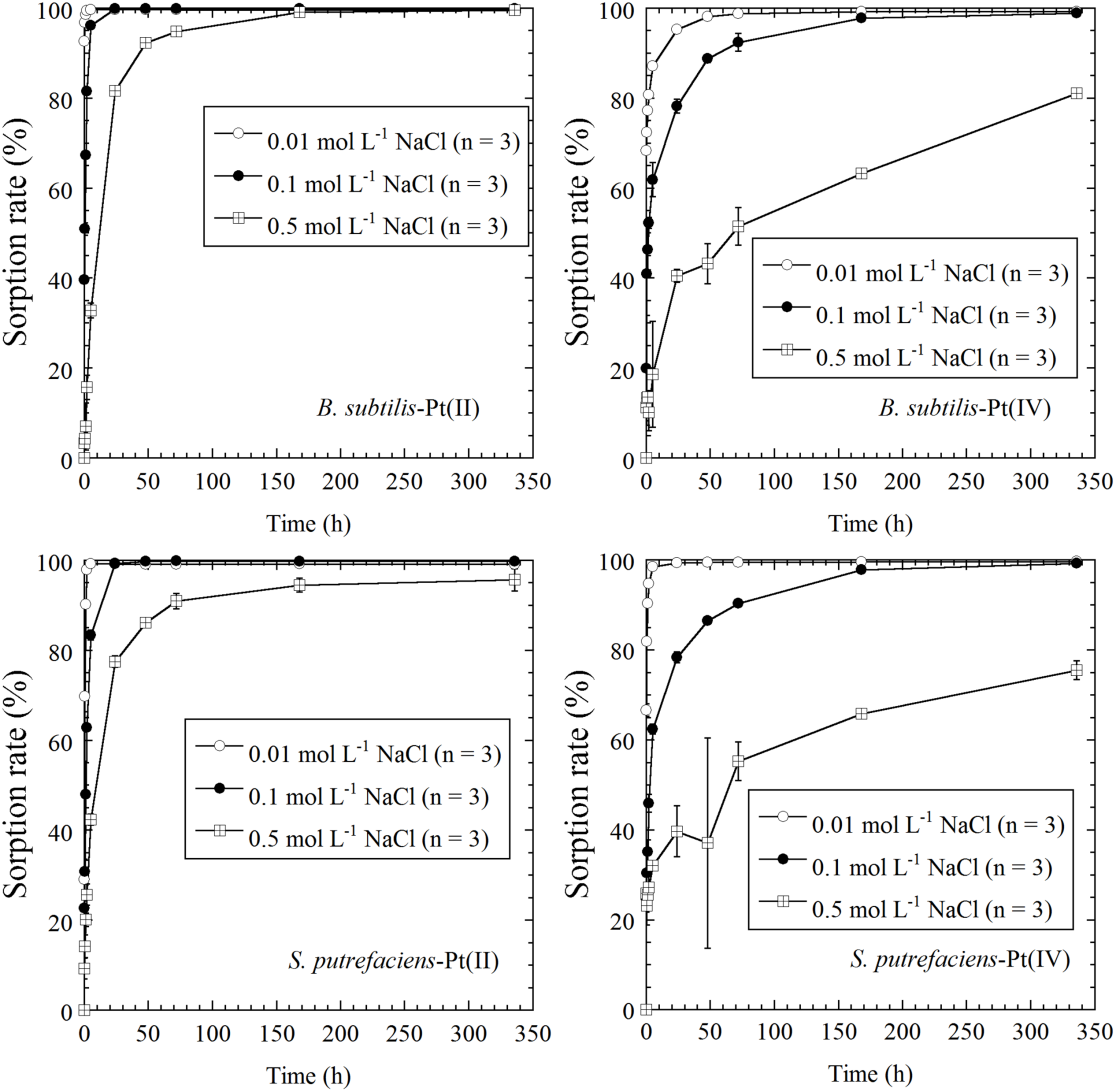
Time-course sorption of Pt(II) and Pt(IV) on *B*. *subtilis* and *S*. *putrefaciens* in solutions with 0.01 mol L^-1^, 0.1 mol L^-1^, and 0.5 mol L^-1^ NaCl at pH 2. The error bars indicate the 1σ value of triplicate experiments.

The results of sorption experiments at pH 2 in solutions with different NaCl concentrations are shown in Fig 2. These experiments were conducted to examine the influence of the concentration of Cl^-^ on the sorption behavior. The sorption behavior of Pt was clearly influenced by increasing the concentration of NaCl. Both *B*. *subtilis* and *S*. *putrefaciens* showed a similar response in the sorption of Pt(II) and Pt(IV) species with changes in the concentration of Cl^-^ ions. Overall, an increase in the concentration of NaCl slowed the sorption of Pt and decreased the concentration of Pt sorbed. As described above, a possible mechanism for sorption is where the negatively charged Pt chloro-complexes of PtCl_4_^2-^ and PtCl_6_^2^ bond to the protonated amine groups on the cell’s surface [15,17]. While higher NaCl (Cl^-^) concentrations promote the formation of Pt chloro-complexes, an increase in the concentration of Cl^-^ ions can lead to a competition at the sorption sites for the limited numbers of amine groups, resulting in a slowed Pt sorption and a decrease in the concentration of Pt sorbed [6,26].

Biosorption is considered to be a metabolism-independent process [6,9]. de Vargas, et al. [6] indicated that autoclaving cells caused little change to their properties as a sorbent and any enzymatic contribution was not significant in biosorption. In our experiments, Pt(II) and Pt(IV) sorption did not decrease after reaching a sorption maximum value at pH 2 and 3, and Pt sorption continued to increase for higher pH and NaCl concentrations (Figs 1 and 2). Even under slightly rigorous conditions (i.e., low pH without any nutrients), bacteria worked well as a sorbent for a period of at least two weeks. Our results also suggest that Pt biosorption is a metabolism-independent process, which occurs at the interface between a cell’s surface and the surrounding solution.

### XANES spectra

**Fig 3:**
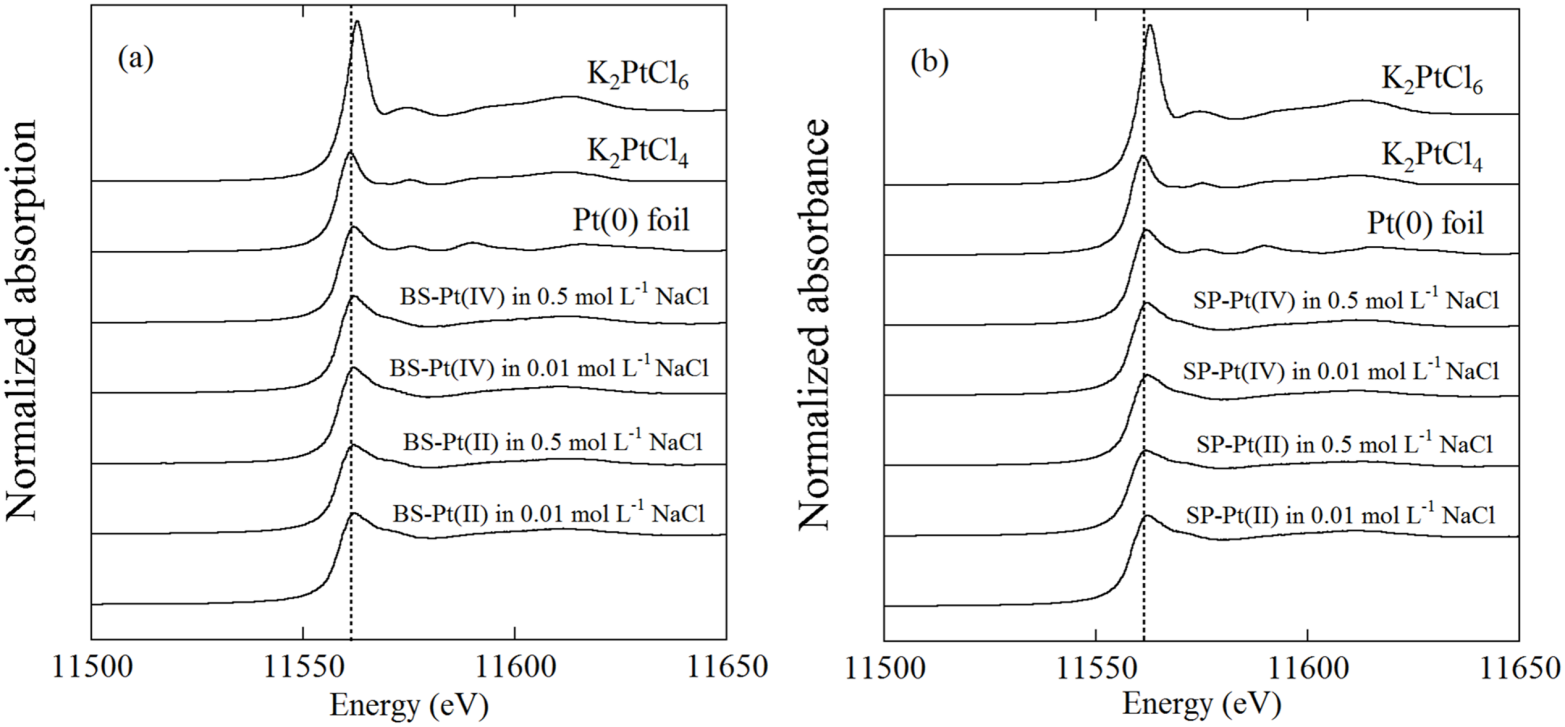
Platinum L_III_-edge XANES spectra of reference materials and Pt-sorbed bacterial cells at pH 2. (a) *B*. *subtilis* and (b) *S*. *putrefaciens*. The dotted lines indicate the maximum peak energy of K_2_PtCl_4_.

Platinum L_III_-edge XANES spectra are shown in Fig 3. The absorption increase occurring at the Pt L_III_-edge corresponds to the 2p_3/2_→5d electronic transitions [27,28]. XANES spectra reflect the oxidation state of a target element. For example, the shift in the peak maximum energy depends on the oxidation state, and this has been successfully applied to distinguish the oxidation state of redox-sensitive elements, such as As, Co, and Mn [19, 29–31]. XANES spectra of Pt metal foil and K_2_PtCl_4_ with Pt in the divalent state indicate a lower energy position of the peak maximum than K_2_PtCl_6_ with Pt in the tetravalent state, but the energy shift is only ~ 1 eV (Fig 3). The ~ 1 eV energy shift observed in the Pt L_III_-edge XANES spectra is not sufficiently large to distinguish between the oxidation states of Pt(0), Pt(II), and Pt(IV). Therefore, we focused on another observable difference between the divalent and tetravalent species, which is seen in the intensity of the white line peaks. The K_2_PtCl_6_ tetravalent Pt compound has a higher peak in the Pt L_III_-edge XANES spectra than has the K_2_PtCl_4_ divalent Pt compound. The intensity of the white line reflects the unoccupied Pt(5d) orbital because the absorption edge corresponds to an electron transition from the 2p_3/2_ to 5d orbitals in the Pt atom [27,28]. The electron configurations of the zero valent, divalent, and tetravalent species are [Xe]4f^14^5d^9^6s^1^, [Xe]4f^14^5d^8^, and [Xe]4f^14^5d^6^, respectively, and the respective number of unoccupied 5d orbitals is 1, 2, and 4. The intensity of the peak maximum is expected to correlate positively with the number of unoccupied 5d orbitals in the reference compound. The K_2_PtCl_6_ tetravalent compound indeed showed a higher peak maximum than the zero valent Pt metal foil and the K_2_PtCl_4_ divalent species (Fig 3).

Measurement of the reference compounds demonstrated that divalent and tetravalent Pt species can be distinguished sufficiently using the intensity of the white-line peaks in the Pt L_III_-edge XANES spectra. The point to be emphasized is that Pt(II)-sorbed and Pt(IV)-sorbed cell samples of *B*. *subtilis* and *S*. *putrefaciens* showed almost identical XANES spectra (Fig 3). This suggests Pt(II) oxidation to Pt(IV) or Pt(IV) reduction to Pt(II) occurs on the cell’s surface after sorption. The intensity of the white-line peaks of the Pt(II)-sorbed and Pt(IV)-sorbed bacterial cells was similar to that of the divalent K_2_PtCl_4_ reference material (Fig 3). The intensity of the white-line peaks of Pt-sorbed cells excluded the presence of Pt(IV) on the cell’s surface, and therefore, a reduction of Pt(IV) species is the more plausible mechanism. Changes in the concentration of NaCl did not affect the XANES spectra, and similar reduction occurred in solutions with different concentrations of NaCl.

It was difficult to distinguish the zero valent and divalent states of Pt using XANES spectra, because the intensities of the white-line peaks for the two oxidation states were similar (Fig 3). Therefore, we must consider the possibility of Pt(II) and Pt(IV) being reduced to Pt(0) on the cell’s surface as well. If such a reduction of the Pt species occurred, then precipitates composed of Pt(0) metal particles on the cell’s surface would be observed [4,5]. However, the possibility of any precipitation of Pt(0) can be excluded from our EXAFS analysis, which did not support the existence of Pt(0), as discussed below.

### EXAFS spectra

**Fig 4:**
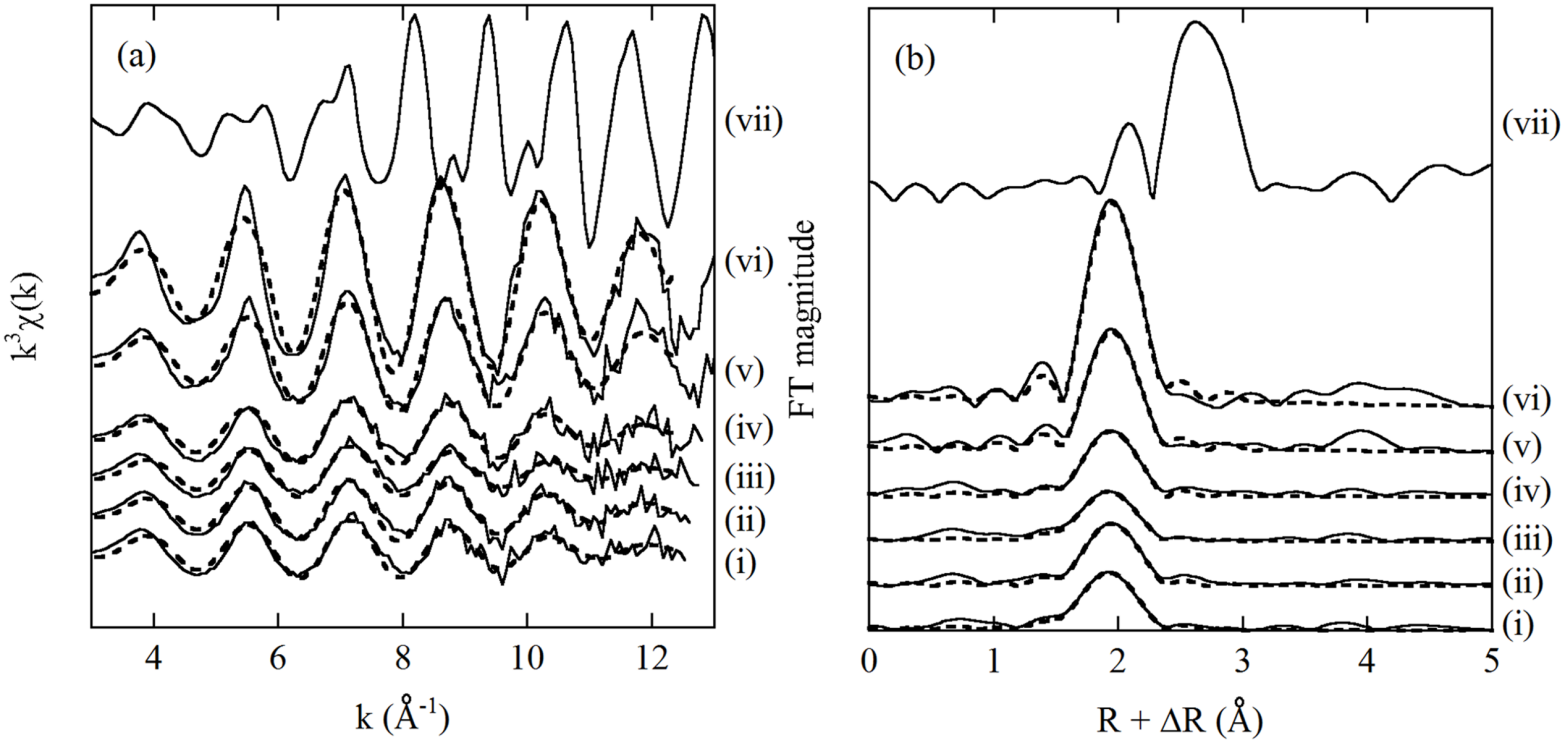
(a) The k^3^-weighted χ(k) functions and (b) RSF values for Pt-sorbed bacterial cells of *B*. *subtilis* at pH 2: (i) Pt(II)-sorbed cell in 0.01 mol L^-1^ NaCl, (ii) Pt(II)-sorbed cell in 0.5 mol L^-1^ NaCl, (iii) Pt(IV)-sorbed cell in 0.01 mol L^-1^ NaCl, (iv) Pt(IV)-sorbed cell in 0.5 mol L^-1^ NaCl, (v) 1000 mg L^-1^ Pt(II) in 1 mol L^-1^ HCl, (vi) 1000 mg L^-1^ Pt(IV) in 1 mol L^-1^ HCl and (vii) Pt metal foil. The dashed lines indicate the fitted results obtained from an EXAFS simulation.

**Fig 5:**
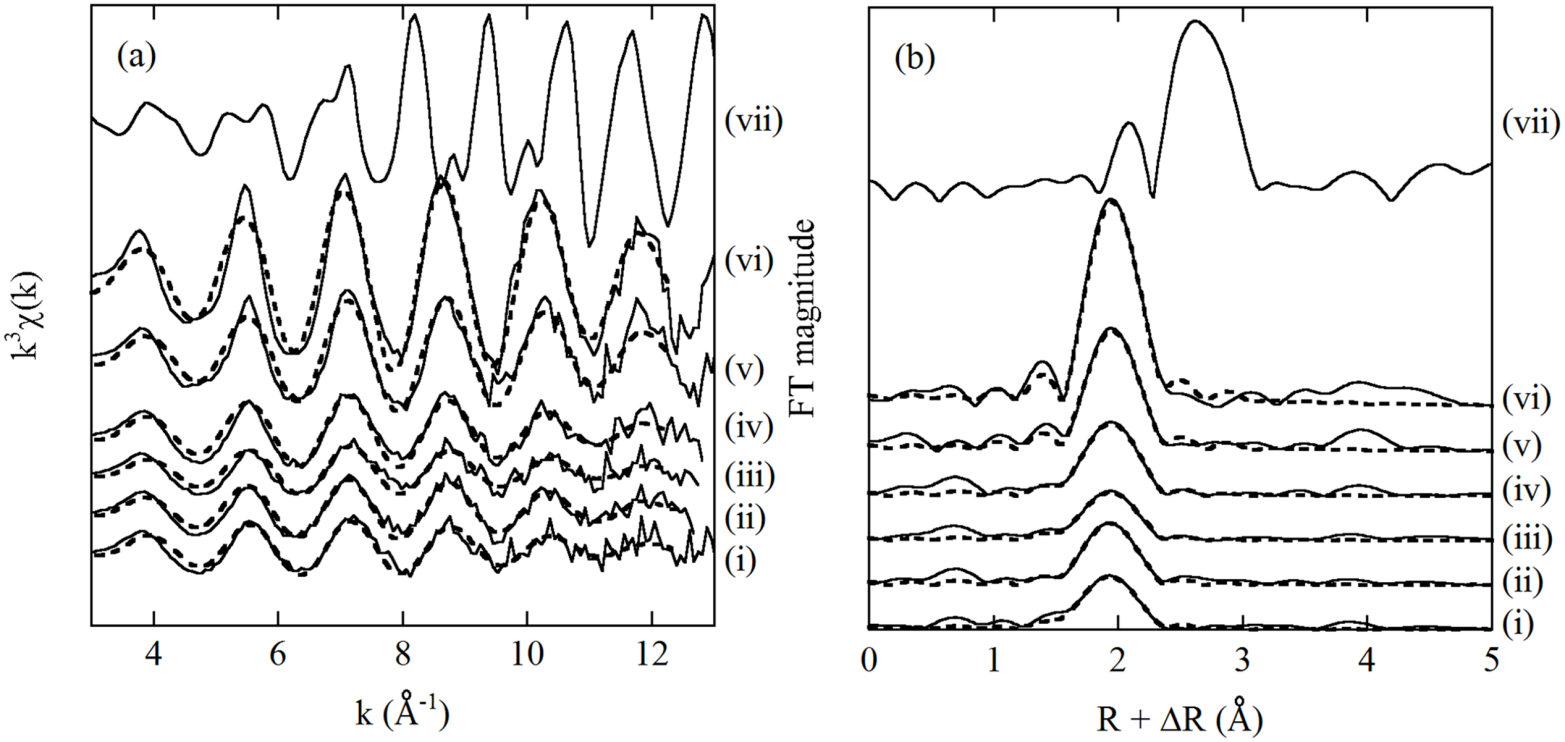
(a) The k^3^-weighted χ(k) functions and (b) RSF values for Pt-sorbed bacterial cells of *S*. *putrefaciens* at pH 2: (i) Pt(II)-sorbed cell in 0.01 mol L^-1^ NaCl, (ii) Pt(II)-sorbed cell in 0.5 mol L^-1^ NaCl, (iii) Pt(IV)-sorbed cell in 0.01 mol L^-1^ NaCl, (iv) Pt(IV)-sorbed cell in 0.5 mol L^-1^ NaCl, (v) 1000 mg L^-1^ Pt(II) in 1 mol L^-1^ HCl, (vi) 1000 mg L^-1^ Pt(IV) in 1 mol L^-1^ HCl and (vii) Pt metal foil. The dashed lines indicate the fitted results obtained from an EXAFS simulation.

The k^3^-weighted χ(k) function and RSF values for Pt-sorbed bacterial cells of *B*. *subtilis* and *S*. *putrefaciens* are shown in Figs 4 and 5, respectively. Plantinum(II) and Pt(IV) in a 1 mol L^-1^ HCl solution and Pt-sorbed bacterial samples both showed similar frequencies of the EXAFS oscillations in k-space (Figs 4a and 5a). The corresponding RSF values showed peaks occurring at R + ΔR = 1.95 Å (Figs 4b and 5b), which were attributed to the first shell of the chlorine atoms. The EXAFS spectra suggest that Pt sorbed on bacterial surface was coordinated by Cl ions, similar to PtCl_4_^2-^ and PtCl_6_^2-^.

The analytical results of the best-fit parameters to the EXAFS spectra are listed in Table 1. The interatomic distances between the Pt and Cl ions in the bacterial samples were almost identical (i.e., 2.30–2.31 Å) to those in the Pt(II) and Pt(IV) solutions (2.31 and 2.32 Å, respectively). It was difficult to distinguish between the Pt(II) and Pt(IV) species from the interatomic distances the between the Pt and Cl ions. However, the CN of the Cl ions is different in PtCl_4_^2-^ and PtCl_6_^2-^. The CN of Cl ions obtained from the EXAFS analysis ranged in value from 3.1 to 4.3 for the bacterial samples (Table 1), which are close to the value for PtCl_4_^2-^. Considering any fitting errors and the accuracy of the EXAFS analysis, the coordination structure of Pt sorbed on the bacterial surface was characterized by Pt-Cl bonds having a fourfold coordination (i.e., PtCl_4_^2-^).

**Table 1:** Structural parameters for Pt(II) and Pt(IV) in 1 mol L^-1^ HCl solution and Pt sorbed on bacterial cells obtained by curve-fitting of EXAFS spectra (CN, coordination number; R, interatomic distance; ΔE_0_ (eV), threshold E_0_ shift; σ, Debye-Waller factor).

	Sample	NaCl concentrration	k range (Å)	Shell	CN	R (Å)	ΔE_0_ (eV)	σ^2^ (x 10^3^ Å^2^)	Residual (%)
*B*. *subtilis*	Pt(II) on BS	0.01 mol L^-1^	3.08–12.32	Pt-Cl	4.0 ± 0.8	2.298 ± 0.013	11.9 ± 2.2	8.8	0.4
	Pt(II) on BS	0.5 mol L^-1^	3.08–12.32	Pt-Cl	3.5 ± 0.7	2.310 ± 0.012	13.1 ± 2.3	7.1	0.4
	Pt(IV) on BS	0.01 mol L^-1^	3.08–12.32	Pt-Cl	3.5 ± 0.7	2.297 ± 0.013	11.9 ± 2.2	9.2	0.6
	Pt(IV) on BS	0.5 mol L^-1^	3.08–12.32	Pt-Cl	3.6 ± 0.7	2.309 ± 0.012	12.3 ± 2.4	6.7	0.2
*S*. *putrefaciens*	Pt(II) on SP	0.01 mol L^-1^	3.08–12.32	Pt-Cl	3.7 ± 0.7	2.299 ± 0.013	13.0 ± 2.2	9.0	0.6
	Pt(II) on SP	0.5 mol L^-1^	3.08–12.32	Pt-Cl	3.3 ± 0.7	2.304 ± 0.012	11.6 ± 2.3	6.7	0.04
	Pt(IV) on SP	0.01 mol L^-1^	3.08–12.32	Pt-Cl	3.1 ± 0.6	2.301 ± 0.012	12.8 ± 2.3	8.3	0.4
	Pt(IV) on SP	0.5 mol L^-1^	3.08–12.32	Pt-Cl	4.3 ± 0.8	2.309 ± 0.012	12.6 ± 2.3	7.4	0.1
Reference	1000 mg L^-1^ Pt(II) in 1 mol L^-1^ HCl		3.08–12.32	Pt-Cl	4	2.314 ± 0.011	9.6 ± 2.5	3.7	0.1
	1000 mg L^-1^ Pt(IV) in 1 mol L^-1^ HCl		2.94–12.18	Pt-Cl	6	2.322 ± 0.011	7.9 ± 2.6	2.8	0.3

Accuracy in the fitted parameters was estimated to be generally ± 0.02 Å for R, ± 20% for CN, and 20% for σ.

Errors attached to the fitting parameters were estimated during simulation of EXAFS spectra.

The coordination numbers of the Pt(II) and Pt(IV) in 1mol L^-1^ HCl were fixed at 4 and 6, respectively, during the fitting of EXAFS spectra.

It should be noted that all the Pt(II)-sorbed and Pt(IV)-sorbed cell samples of *B*. *subtilis* and *S*. *putrefaciens* showed almost identical EXAFS spectra in both k- and R-spaces (Figs 4 and 5), resulting in similar fitting results (Table 1). Similar to the XANES spectra (Fig 3), the EXAFS spectra suggest Pt(IV) was reduced to Pt(II) on the bacterial cell surface. Changes in the concentration of NaCl did not influence the coordination structures of Pt on the bacterial cells, possibly because Cl^-^ only acted as a competitor for Pt in the biosorption process.

The EXAFS oscillations of Pt metal foil were quite different from those observed for the Pt solutions and Pt-sorbed bacterial cell samples (Figs 4a and 5a). In addition, Pt metal foil showed two distinct peaks occurring at R + ΔR = 2.1 and 2.6 Å (Figs 4b and 5b). These characteristics of Pt metal allowed us to determine the existence of Pt(0) metal particles on the cell’s surface. However, we did not find a contribution from Pt(0) on the bacterial cells in the EXAFS spectra. The EXAFS spectra demonstrated that Pt(II) was not reduced to Pt(0) on the bacterial cell surface. Also, high resolution TEM analysis would help to confirm the existence of Pt(0) on cell surface.

### Mechanism of Pt biosorption on bacterial cell surfaces

We carried out simple sorption experiments of Pt(II) and Pt(IV) on bacterial cells without using any organic compounds as electron donors. Nevertheless, the XANES and EXAFS spectra indicated that Pt(IV) was reduced to Pt(II) on the cell surface (Figs 3, 4, and 5). Similarly, previous work has pointed out the possibility of a reduction of Au(I) and Pd(II) to the metallic state (i.e., Au(0) and Pd(0)) on biomass in the absence of organic materials as an exogenous electron donor [6,32]. An investigation into what is a suitable electron donor for the reduction of Pt(IV) is an interesting and important issue, but it is beyond the scope of this study.

As mentioned above, the mechanism for Pt biosorption is not fully understood, and details of the coordination structure of Pt on the cell’s surface are still not clear because of the scarcity of spectroscopic studies. We have demonstrated that Pt sorbed on a bacterial cell surface is present as a Pt(II) chloro-complex, possibly PtCl_4_^2-^, using XANES and EXAFS (Figs 3, 4, and 5). The results of our XAFS analysis are consistent with the fact that Pt biosorption is favored at lower pH in NaCl solutions (Fig 1), where anionic species of the Pt chloro-complex are possibly sorbed onto protonated amine groups on the cell wall [15,17].

de Vargas et al. [6] conducted biosorption experiments using Pd and Pt on three bacterial species: *Desulfovibrio desulfuricans*, *D*. *vulgaris*, and *D*. *fructosivorans*, which are all Gram-negative bacteria. These three species in the same genus showed different sorption capacities for Pd and Pt, but their response to changes in the composition of the solutions were similar to one another. Similarly, the results of this study indicate that *B*. *subtilis* and *S*. *putrefaciens* showed similar trends to changes in the concentration of the NaCl solution and pH, suggesting that a similar mechanism governs the biosorption of Pt (Figs 1 and 2). The differences observed between different bacterial species with respect to the maximum sorption capacity are probably related to the differences in the exact composition of the cell wall, namely, the available number of binding sites. The XANES and EXAFS spectra indicated that the biosorption mechanism of Pt on *B*. *subtilis* (a Gram-positive bacterium) and *S*. *putrefaciens* (a Gram-negative bacterium) was almost identical for both (Figs 3, 4, 5, and Table 1), although they have different cell wall structures and functional compositions. The direction of the results observed in this study indicates that sorption of negatively charged Pt chloro-complexes onto the protonated amine groups on a cell’s surface is a common process, which is of fundamental importance for understanding Pt biosorption [15,17].

## Conclusions

We have investigated the coordination structures of Pt(II) and Pt(IV) sorbed on bacterial cells in NaCl solutions. XANES spectra suggested that Pt(IV) was reduced to Pt(II) on the cell’s surface, which was supported by an EXAFS analysis. EXAFS analysis also demonstrated that the coordination structure of Pt sorbed on the bacterial cells was via a chloro-complex with a fourfold coordination, and was possibly PtCl_4_^2-^. Both *B*. *subtilis* and *S*. *putrefaciens* showed almost identical coordination structures, indicating that sorption of negatively charged Pt chloro-complexes onto the protonated amine groups on the cell’s surface is an essential process for Pt biosorption.
